# Harnessing the Power of Complementarity Between Smart Tracking Technology and Associated Health Information Technologies: Longitudinal Study

**DOI:** 10.2196/51198

**Published:** 2024-10-01

**Authors:** Youyou Tao, Ruilin Zhu, Dezhi Wu

**Affiliations:** 1 Department of Information Systems and Business Analytics College of Business Administration Loyola Marymount University Los Angeles, CA United States; 2 Department of Management Science Lancaster University Lancaster United Kingdom; 3 Department of Integrated Information Technology Molinaroli College of Engineering and Computing University of South Carolina Columbia, SC United States

**Keywords:** health IT, smart tracking technology, mobile IT, health information exchange, electronic health record, readmission risk, complementarity effects, mobile phone

## Abstract

**Background:**

Smart tracking technology (STT) that was applied for clinical use has the potential to reduce 30-day all-cause readmission risk through streamlining clinical workflows with improved accuracy, mobility, and efficiency. However, previously published literature has inadequately addressed the joint effects of STT for clinical use and its complementary health ITs (HITs) in this context. Furthermore, while previous studies have discussed the symbiotic and pooled complementarity effects among different HITs, there is a lack of evidence-based research specifically examining the complementarity effects between STT for clinical use and other relevant HITs.

**Objective:**

Through a complementarity theory lens, this study aims to examine the joint effects of STT for clinical use and 3 relevant HITs on 30-day all-cause readmission risk. These HITs are STT for supply chain management, mobile IT, and health information exchange (HIE). Specifically, this study examines whether the pooled complementarity effect exists between STT for clinical use and STT for supply chain management, and whether symbiotic complementarity effects exist between STT for clinical use and mobile IT and between STT for clinical use and HIE.

**Methods:**

This study uses a longitudinal in-patient dataset, including 879,122 in-patient hospital admissions for 347,949 patients in 61 hospitals located in Florida and New York in the United States, from 2014 to 2015. Logistic regression was applied to assess the effect of HITs on readmission risks. Time and hospital fixed effects were controlled in the regression model. Robust standard errors (SEs) were used to account for potential heteroskedasticity. These errors were further clustered at the patient level to consider possible correlations within the patient groups.

**Results:**

The interaction between STT for clinical use and STT for supply chain management, mobile IT, and HIE was negatively associated with 30-day readmission risk, with coefficients of –0.0352 (*P*=.003), –0.0520 (*P*<.001), and –0.0216 (*P*=.04), respectively. These results indicate that the pooled complementarity effect exists between STT for clinical use and STT for supply chain management, and symbiotic complementarity effects exist between STT for clinical use and mobile IT and between STT for clinical use and HIE. Furthermore, the joint effects of these HITs varied depending on the hospital affiliation and patients’ disease types.

**Conclusions:**

Our results reveal that while individual HIT implementations have varying impacts on 30-day readmission risk, their joint effects are often associated with a reduction in 30-day readmission risk. This study substantially contributes to HIT value literature by quantifying the complementarity effects among 4 different types of HITs: STT for clinical use, STT for supply chain management, mobile IT, and HIE. It further offers practical implications for hospitals to maximize the benefits of their complementary HITs in reducing the 30-day readmission risk in their respective care scenarios.

## Introduction

### Background

Substantial health expenditure has been a perennial issue for most hospitals in the United States [1,2]. In 2022, health care expenditure in the United States reached US $4.5 trillion or US $13,493 per capita, which constituted 17.3% of the gross domestic product of the United States [3]. Hospital readmission has been identified as a key contributor to this high expenditure [4-6], with preventable readmissions after hospital discharge estimated to cost approximately US $25 billion every year [5-7].

In response to the challenge of excessive health expenditure, hospitals have adopted health ITs (HITs) to improve hospital operation efficiency and clinical quality [8-11]. HIT is an umbrella term that includes various IT applications that are used in a health care setting [12-14], such as smart tracking technology (STT), health information exchanges (HIE), and mobile IT apps [12-14].

STT has been widely applied at points of care to facilitate clinical workflows among health care providers and business sectors [15-18]. In a hospital setting, STT primarily incorporates radio frequency identification and barcode technologies [17,18], which are supposed to be complementary and embedded in the existing HIT infrastructure. STT enables a novel IT structure to streamline clinical processes and facilitate the management of patients and assets [15,16]. The value–based purchasing program, a federal regulation, alters hospital reimbursements from a fee-for-service model to a value-based model, a shift that has further emphasized the substantial need for accurate tracking of resources used in care delivery and health outcomes [19,20]. Therefore, in this study, we assessed the impact of STT for clinical use on patient outcomes.

Previous research has demonstrated the positive effects of STT, such as reducing medical errors and readmission rates and increasing patient satisfaction [19,21], in clinical settings. However, the effects of potential synergy between STT and other complementary HIT functions have not been empirically investigated with a large sample. Although Zhu et al [17] explored the factors influencing the adoption of STT for clinical use and supply chain use, they did not examine the joint and complementary effects of these 2 technologies on health outcomes. At the peak of the COVID-19 pandemic, a few studies examined the application of radio frequency identification and mobile IT for real-time contact tracing [22,23], but none of the studies have examined their joint effects in a hospital setting. Bradley et al [19] conducted a study to assess the effect of the combined use of STT and electronic data interchange on hospital performance. However, their research had 2 practical limitations: 1) they only included 2 technologies (STT and electronic data interchange) in their technology bundle, and 2) they only considered the 2 conditions, heart failure and pneumonia, as their readmission measures, disregarding other conditions. Theoretically, their research did not consider the nature of joint effects because different complementarities can coexist in a single system [24]. Therefore, this study seeks to address this knowledge gap by broadening the scope of the joint effects of STT for clinical use and other HITs on all-cause readmissions as well as by exploring the nuanced impacts on individuals with various disease types, including both chronic and acute conditions.

From an information systems (IS) perspective, the complexity of HITs is reflected by its chain of various embedded systems and technologies used to meet the diverse needs of health care providers, patients, and hospitals across interconnected units [11,19,24-26]. By understanding the intricate relationships among various HITs, researchers and practitioners can gain a comprehensive understanding of their complexity. This includes insights into the integration, interoperability, and operational challenges of different HITs. This can further lead to optimal resource allocations, improved patient care integration, increased hospital efficiency, and enhanced clinical quality. However, the current scarcity of available literature on the joint effects of STT for clinical use and other HITs on patient outcomes indicates a significant opportunity to advance our understanding of synergistic effects of HITs on health outcomes [25]. In this study, to address the high patient readmission rates in the United States, we aimed to explore the joint effects of STT for clinical use and other complementary HIT functionalities on the risk of 30-day all-cause readmission within a hospital setting.

To examine the joint effects of STT for clinical use and other HITs, we adopted the complementarity theory. This theory suggests that the joint use of multiple technologies can potentially lead to a greater overall effect than the sum of their individual effects [27]. Two distinct types of complementarity effects have been identified [27,28]: (1) pooled complementarity, which emerges when similar resources are applied across different application domains, and (2) symbiotic complementarity, which occurs when different resources are used within the same application domain [25,28].

### Objective

**Figure 1:**
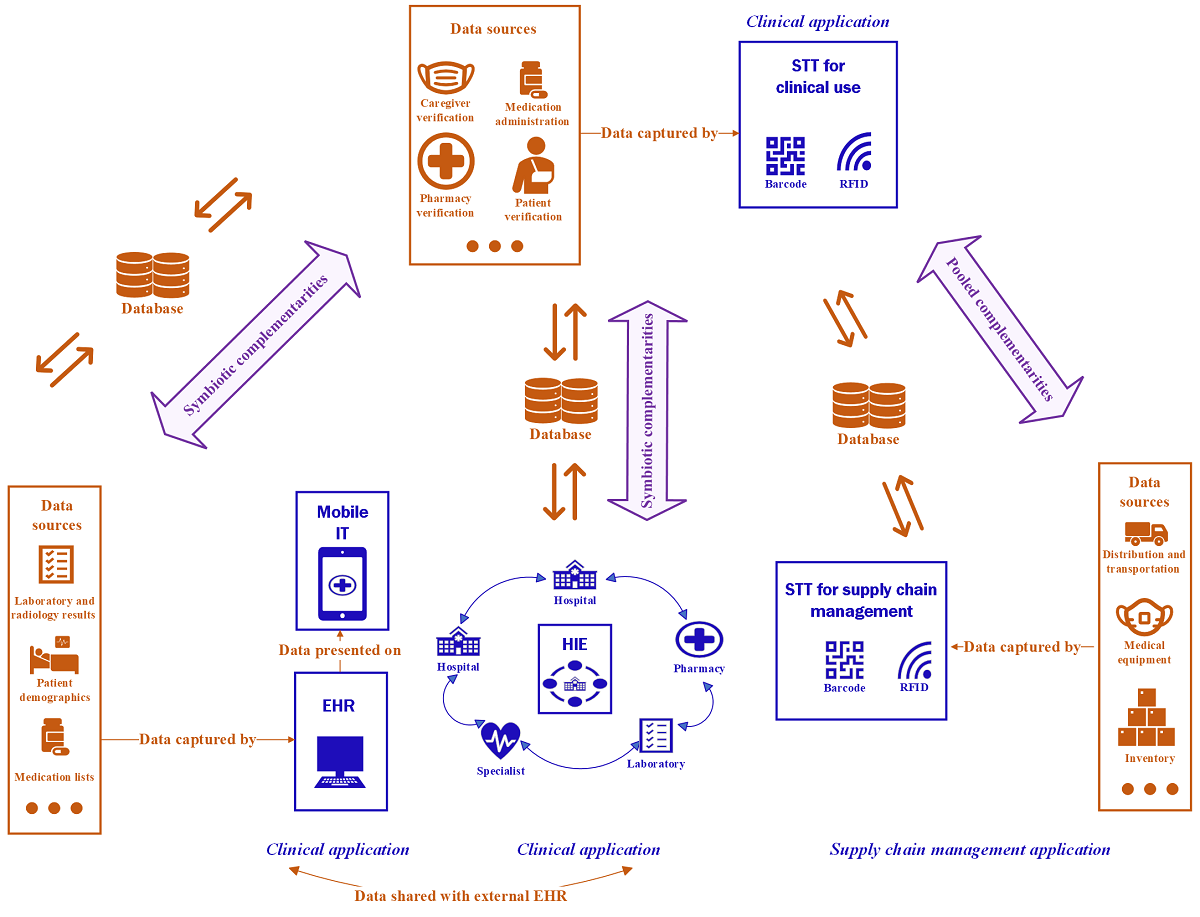
Use case examples for the complementarity effects among 4 health ITs (HITs): smart tracking technology (STT) for clinical use, STT for supply chain management, mobile IT, and health information exchange (HIE). EHR: electronic health record.

This study aimed to investigate both pooled and symbiotic complementarities. To illustrate the complementarity effects among HITs studied in this research, we presented the use case examples shown in Figure 1. In the following paragraphs, we will first discuss the pooled complementarity effects and then discuss symbiotic complementarity effects. Pooled complementarities may exist between 2 distinct applications of STT: one used in a clinical context and the other used for supply chain management. Although both applications are based on the same technology, they are implemented in different functional areas. STT for clinical use is used to track patients and medications in clinical settings, whereas STT for supply chain management is used to facilitate operations and track products within the supply chain management chain at a hospital setting [17]. When the aforementioned 2 STTs are implemented together, it can lead to more efficient workflows and operations across the entire hospital. For instance, an integrated STT system can provide end-to-end visibility and tracking capability from the points of medication, supplies, and equipment to their use and can also help with the patient’s bedside monitoring [21]. This can reduce medical errors and hospital waste as well as ensure quick and accurate identification of patients and tracking of their medications, supplies, and equipment, resulting in improved patient safety, clinical outcomes, and operational efficiency [21].

Next, the researchers investigated the symbiotic complementarity between STT for clinical use and mobile IT. The researchers also investigated the symbiotic complementarity between STT for clinical use and HIE. The adoption of mobile technologies such as smartphones and tablets has become increasingly prevalent within health care institutions, particularly when combined with electronic health records (EHRs) to facilitate the clinical workflows of health care providers [29]. The combination of STT and mobile IT has been implemented in in-patient settings to improve patient identification, match correct medications and treatments with patients, and access up-to-date integrated clinical and medication information. Previous studies have demonstrated that the implementation of STT led to a decrease in adverse drug events and medication errors, which are often caused by incomplete patient information, inadequate tracking of patients and medications, and insufficient communication between disparate HITs [30-32]. Thus, it is important to examine the symbiotic complementarities between STT for clinical use and mobile IT. This is because these 2 unique HITs jointly contribute to the capturing and tracking of clinical data at the point of care.

Furthermore, while STT for clinical use facilitates the capturing and processing of data at the point of care, HIE enables and enhances the sharing of quality and efficiency of patient health information across different health care organizations, laboratories, and specialists [33-35]. HIE facilitates information exchange among hospitals, laboratories, and specialists as patients seek continuity of care, which is crucial during patient transitions between different health care facilities [34,35]. For patients with multiple admissions, it is vital to have their complete clinical information and historical records accessible during each admission. The effective use of STT can streamline the information flow and reduce errors, hospital waste, and delays in clinical processes [36]. When STT is integrated with HIE, the flow of information may be further enhanced in terms of data accuracy and completeness. Therefore, this study also aims to investigate the symbiotic complementarity between STT for clinical use and HIE. Though these are 2 distinct HITs, both are essential for clinical tracking and for capturing and sharing of data in the clinical setting.

Moreover, the structure of hospitals and the type of disease can lead to heterogeneous effects of HITs [25,34,37]. For instance, hospitals affiliated with multihospital health care systems have been found to have more resources and capabilities to effectively implement and coordinate different HITs [17]. Conversely, unaffiliated, individual hospitals may experience challenges such as lack of financial and human resources, making the implementation and effective use of HITs more difficult. Further, in terms of disease types, certain conditions might benefit more from HIT than others. For example, patients with chronic conditions that usually last >12 months and often require long-term patient monitoring and continuous care management may benefit from the joint use of HITs due to the ability to constantly update, access, and analyze patient data. This can lead to improved health outcomes [6,25,34]. In contrast, patients with acute conditions that require immediate intervention may benefit from HITs due to their speed and accuracy in processing information, allowing for prompt diagnosis and treatment [25,34]. Thus, this study also explores how hospital affiliations and the patients’ primary diagnoses impact the joint effects.

## Methods

### Data Collection and Sample

This is a retrospective study and our data consisted of patient admission records from 61 hospitals in New York and Florida in the United States across 2 years, 2014 and 2015. The unit of analysis in this study was patient-admission level. The researchers collected and integrated data from 3 different sources. First, this research used data from the Healthcare Cost and Utilization Project (HCUP)’s state in-patient datasets to obtain patient characteristics, admission, clinical, and billing information [38]. Second, the researchers applied data from the American Hospital Association’s (AHA’s) annual surveys to obtain hospital characteristic data [39]. Third, the researchers used the AHA’s IT supplement files for the 2013 to 2014 period to obtain HIT implementation data [39]. Given the lagged effects of HIT adoption, this research followed the method that has been widely adopted to map datasets with HIT variables lagged for 1 year [25,40-42]. These 3 datasets were merged by matching hospital identification from HCUP and AHA. In this study, the researchers excluded 2 categories of patient admissions. First, those related to pregnancy, childbirth, and the puerperium, as the patterns of readmission within this group can significantly differ from others. Second, we left out the admissions of patients who were <18 years old, given that their treatment approaches and readmission patterns frequently vary from those observed in adults [43]. In addition, we also excluded patients with a single admission and those whose first admission date was in the previous year (2015) to ensure that the follow-up time and observation time (1 year) were consistent across the datasets. The final sample comprised 879,122 in-patient admissions for 347,949 patients across 61 hospitals.

### Variable Description

To investigate joint HIT impacts on health outcomes, we focused on the risk of 30-day all-cause readmission, which is measured as a binary variable, indicating whether a patient’s hospital admission was followed by at least one 30-day hospital readmission.

This study examined 4 HITs: STT for clinical use, STT for supply chain use, mobile IT, and HIE. STT for clinical use measures the level of STT implementation in hospitals for clinical use, including tracking technologies for medication administration, patient verification, caregiver verification, and pharmacy verification. STT for supply chain use measures whether the hospital has implemented STT for supply chain management. Mobile IT measures whether the hospital has fully implemented mobile devices, such as tablets and smartphones, to connect to EHRs. HIE measures whether the hospital has fully implemented HIE systems. The researchers used a variety of methods to construct these variables. Specifically, the researchers constructed STT for clinical use by counting the number of tracking technologies that were completely implemented at a hospital, a common approach used in previous studies in both the IS and health informatics literature [17,42,44]. Consequently, the STT for clinical use variable ranged from 0 to 4, with 0 indicating that the hospital had not implemented any of the four tracking technologies and 4 indicating that the hospital had implemented all four tracking technologies (tracking technologies for medication administration, patient verification, caregiver verification, and pharmacy verification). The researchers constructed STT for supply chain use as a binary variable, with 1 indicating full implementation and 0 otherwise. Mobile IT and HIE were also constructed as binary variables, with 1 indicating implementation and 0 indicating otherwise.

Hospital-level and patient admission-level variables were included as control variables to account for other potential factors that may affect health outcomes. Hospital-level variables included EHR implementation level (Multimedia Appendix 1), hospital bed size, teaching status, profit status, hospital affiliation (whether the hospital is affiliated to the health care system or not), hospital location (whether the hospital is located in a metropolitan city or not), and state indicator (whether the hospital is located in Florida or New York). The admission-level variables of a patient included gender; race; age; insurance types, including Medicare, Medicaid, and private insurance; the total number of comorbidities; chronic diagnoses; diagnoses; and procedures at current admission. We also included several binary variables. One binary variable indicates whether the primary diagnosis during the current admission was a chronic condition. The definition of a chronic condition was adopted from HCUP in this study. HCUP defines a chronic condition as one persisting for ≥12 months; this either limits self-care, independent living, and social interactions, or requires continuous intervention with medical products, services, and special equipment [45]. Another binary variable denotes whether the admission was emergency or urgent, and a third reflects whether the primary diagnosis fell within the same body system as the previous admission. Furthermore, the study included the frequency of hospital visits since the patient’s first visit and the interval between the current and previous visit. The study also employed 17 different types of body systems from HCUP as dummy variables, including (1) certain conditions originating in the perinatal period; (2) congenital anomalies; (3) diseases of blood and blood-forming organs; (4) diseases of the circulatory system; (5) diseases of the digestive system; (6) diseases of the genitourinary system; (7) diseases of the musculoskeletal system; (8) diseases of the nervous system and sense organs; (9) diseases of the respiratory system; (10) diseases of the skin and subcutaneous tissue; (11) endocrine, nutritional, and metabolic diseases and immunity disorders; (12) factors influencing health conditions and contact with health services; (13) injury and poisoning; (14) infectious and parasitic disease; (15) mental disorders; (16) neoplasms; and (17) symptoms, signs, and ill-defined conditions. Multimedia Appendix 2 presents the summary of statistical variables applied in this study.

### Statistical Analysis

Logistic regression models have been widely used in the readmission literature to estimate the probability of patients being readmitted to hospitals within a certain timeframe [10,46]. Therefore, in this study, logistic regression models were applied to assess the impact of HITs on the 30-day readmission risk. This model accounted for both hospital and year fixed effects. Robust SEs were applied to account for potential heteroskedasticity. The errors were further clustered at the patient level to account for possible within-patient correlations. The model specifications are presented in Multimedia Appendix 3. We applied Stata (version 16; StataCorp) as our analysis software.

### Ethical Considerations

According to the HCUP website [38], “HCUP databases are limited data sets. HCUP databases conform to the definition of a limited data set. A limited data set is health care data in which 16 direct identifiers, specified in the Privacy Rule, have been removed. As per the Health Insurance Portability and Accountability Act, review by an institutional review board (IRB) is not required for the use of limited data sets.” The Loyola Marymount University’s IRB decided to exempt this study from the IRB review because data from AHA’s annual surveys and IT supplement files were collected at the hospital level and did not involve human participants. In addition, the first author of this paper has obtained permission to use the HCUP data, AHA’s annual surveys, and AHA’s IT supplement files stated in this study for analysis and publication.

## Results

### Effects of STT for Clinical Use on 30-Day Readmission Risk

The results estimated from logistic regression models (equations 1 to 4 in Multimedia Appendix 3) are presented in Table 1. Column 1 presents the main effects of HITs on the risk of 30-day readmission. We found that an increase of 1 level in STT implementation for clinical use is associated with a decrease in 30-day readmission risk (β=–.0153; *P*=.004), while an increase of 1 level in STT implementation for supply chain management is associated with an increase in 30-day readmission risk (β=.0485; *P*=.004). Mobile IT showed no significant main effects. A previous study suggested that the effect of HITs implementation on readmission rates is time dependent [19]. It is important to note that because this study relies on only 2 years of retrospective data, the long-term effects of STT on supply chain management and mobile IT may differ from the findings presented here. We also found that HIE implementation is associated with an increase in readmission risk (β=.0499; *P*=.02). Recent studies have yielded mixed results concerning the relationship between HIE implementation and hospital readmission rates. A few studies have suggested that HIE implementation can lead to a decrease in 30-day readmissions [47,48], whereas other studies have indicated no significant effect [49,50] or even an increase in readmission rates [51]. It is possible that HIE implementation may help to identify additional readmissions that were previously missed due to incomplete patient self-reports or a lack of follow-up [51].

**Table 1:** Effects of smart tracking technology (STT) for clinical use on 30-day readmission risk^a^. Logistic regression was applied to assess the impact of health ITs (HITs) on 30-day readmission risk. The data sample included 879,122 in-patient admissions in the states of New York and Florida in the United States between 2014 and 2015. Both chronic and acute disease types are included in the sample.

Variable	1			2				3			4	
	Value	*P* value	Value			*P* value	β		*P* value	Value		*P* value
STT_Clinical_, β (SE)	–.0153^b^ (0.005)	.004	–.0079 (0.006)			.17	.0060 (0.007)		.37	.0012 (0.010)		.91
STT_Supply Chain_, β (SE)	.0485^b^ (0.017)	.004	.1475^c^ (0.038)			<.001	0.0217 (0.018)		.23	.0524^b^ (0.017)		.002
Mobile IT, β (SE)	–.0151 (0.020)	.45	–.0277 (0.020)			.17	.1388^c^ (0.038)		<.001	–.0158 (0.020)		.43
HIE^d^, β (SE)	.0499^e^ (0.021)	.02	.0382^f^ (0.021)			.07	.0439^e^ (0.021)		.04	.1040^b^ (0.034)		.002
STT_Clinical_×STT_Supply Chain_, β (SE)	—^g^	—	–.0352^b^ (0.012)			.003	—		—	—		—
STT_Clinical_×mobile IT, β (SE)	—	—	—			—	–.0520^c^ (0.011)		<.001	—		—
STT_Clinical_×HIE, β (SE)	—	—	—			—	—		—	–.0216^e^ (0.011)		.04
Hospital control	Yes	—	Yes		—		Yes		—	Yes		—
Patient control	Yes	—	Yes		—		Yes		—	Yes		—
Time and hospital fixed effects	Yes	—	Yes		—		Yes		—	Yes		—
Observations, n	879,122	—	879,122		—		879,122		—	879,122		—
Patients, n	347,949	—	347,949		—		347,949		—	347,949		—

^a^The constant has been included. Robust SEs were applied, and the errors were further clustered at the patient level to account for possible within-patient correlations.

^b^*P*<.01.

^c^*P*<.001.

^d^HIE: health information exchange.

^e^*P*<.05.

^f^*P*<.10.

^g^Not available.

Columns 2 to 4 display the interaction effects of HITs. Column 2 shows that the interaction between STT for clinical use and for supply chain management is associated with a significant decrease in readmission risk (β=–.0352; *P*=.003). This translates to a reduction in readmission risk by approximately 3.46% for each level of increase in the interaction effect. This result also indicates a *pooled complementarity* effect due to similar applications in different domains, specifically clinical use and supply chain management. Column 3 shows that the interaction of STT for clinical use and mobile IT is also associated with a lower readmission risk (β=–0.0520; *P*<.001); this translates to a reduction in readmission risk by approximately 5.06% for each level of increase in the interaction effect. This also signifies a *symbiotic complementarity* effect because both of these unique HITs contribute to clinical capturing and tracking at the point of care. Finally, in column (4), the interaction between STT for clinical use and HIE is associated with a lower readmission risk (β=–.0216; *P*=.04); this translates to a reduction in readmission risk by approximately 2.14% for each of level increase in the interaction effect. This result also suggests a *symbiotic complementarity* effect. This is because though these are different HITs, both can be applied in the context of clinical tracking and data capturing and sharing.

**Figure 2:**
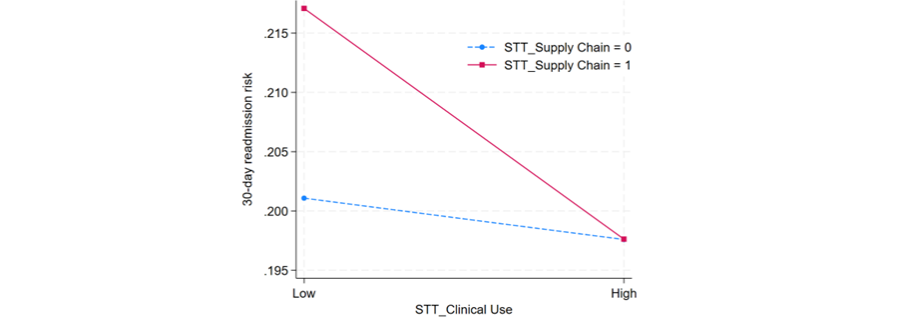
Interaction plot between smart tracking technology (STT) for clinical use and STT for supply chain management. The data sample included 879,122 in-patient admissions in the states of New York and Florida in the United States across 2014 and 2015. Both chronic and acute disease types are included in the sample.

**Figure 3:**
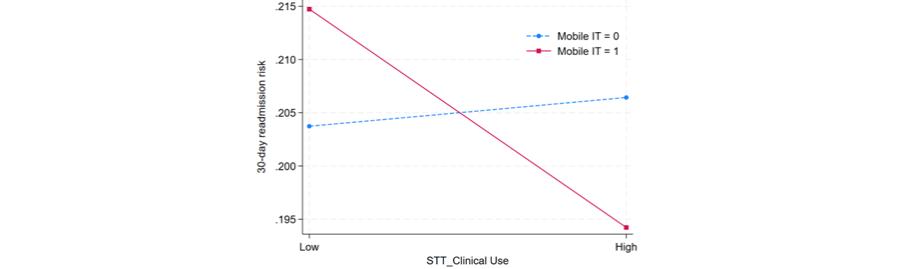
Interaction plot between smart tracking technology (STT) for clinical use and mobile IT. The data sample included 879,122 in-patient admissions in the states of New York and Florida in the United States across 2014 and 2015. Both chronic and acute disease types are included in the sample.

**Figure 4:**
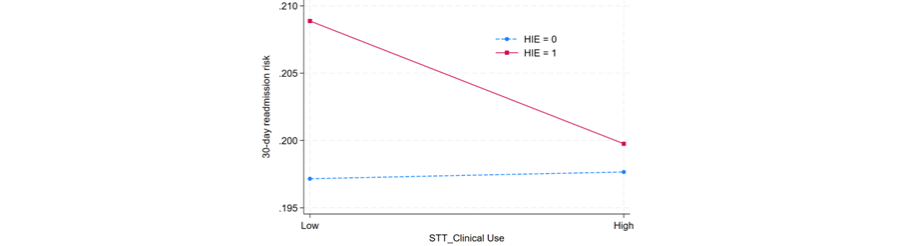
Interaction plot between smart tracking technology (STT) for clinical use and health information exchange (HIE). The data sample included 879,122 in-patient admissions in the states of New York and Florida in the United States across 2014 and 2015. Both chronic and acute disease types are included in the sample.

To better understand the joint effects of STT for clinical use and associated complementary HITs, the researchers further examined the significant 2-way interactions between STT for clinical use and that for supply chain management, between STT for clinical use and mobile IT, and between STT for clinical use and HIE. These interactions are presented in Figures 2-4. In these figures, a high level denotes 1 SD above the mean, and a low level signifies 1 SD below the mean.

Figure 2 illustrates that when hospitals do not implement STT for supply chain management, an increase in STT for clinical use implementation level is associated with a slight decrease in 30-day readmission risk. In contrast, when hospitals implemented STT for supply chain management, an increase in STT for clinical use implementation level is associated with a dramatic reduction in the 30-day readmission risk. Therefore, it suggests that patients admitted to hospitals that have implemented STT for supply chain management experience a more pronounced decrease in the 30-day readmission risk compared with those admitted in hospitals without STT for supply chain management. This result further proves the pooled complementarity effect.

Figure 3 reveals that when mobile IT is not implemented, an increase in STT for clinical use implementation level is associated with an increase in the 30-day readmission risk. In contrast, when hospitals implemented mobile IT, an increase in STT for clinical use implementation level is associated with a lower 30-day readmission risk. This result shows the symbiotic complementarity effect; STT for clinical use reduces the 30-day readmission risk when mobile IT is implemented; however, when mobile IT is not implemented, this risk is slightly increased even when STT for clinical use is implemented.

Figure 4 shows that, in the absence of HIE, an increase in STT for clinical use implementation level slightly increases the 30-day readmission risk. However, with HIE implemented, an increase in STT for clinical use implementation level is associated with a lower 30-day readmission risk. This outcome further provides evidence for the symbiotic complementarity effect; STT for clinical use reduces the 30-day readmission risk when HIE is implemented; however, when HIE is not implemented, this risk is slightly increased even when STT for clinical use is administered.

### The Role of Hospital Affiliation and Disease Type on the Joint Effects

Next, we evaluate how the joint effects between STT for clinical use and that for supply chain management, between STT for clinical use and mobile IT, and between STT for clinical use and HIE differ among hospitals with different affiliations, as well as between patients with chronic or acute diseases. Table 2 presents the results regarding the influence of hospital affiliation on these joint effects. From this table, we identify that for hospitals within the health system, both the interaction effect between STT for clinical use and that for supply chain management and the interaction effect between STT for clinical use and mobile IT were associated with a decrease in 30-day readmission. For hospitals not within the health system, the interaction effect between STT for clinical use and that for supply chain management was associated with a higher 30-day readmission risk. This finding aligns with the earlier research suggesting that health system–affiliated hospitals, with their better resources and capabilities, can implement and coordinate different HITs more effectively [17], resulting in improved clinical outcomes following HIT implementation.

**Table 2:** The role of hospital affiliation on the effects of smart tracking technology (STT) for clinical use^a^. Logistic regression was applied to assess the impact of health ITs (HITs) on the 30-day readmission risk. The data sample in models 1 to 3 included 662,874 in-patient admissions and models 4 to 6 included 186,496 in-patient admissions in the states of New York and Florida in the United States across years 2014 and 2015. Both chronic and acute disease types are included in the sample.

Variable	In the health system									Not in the health system							
	1			2			3			4			5			6	
	Value	*P* value	Value		*P* value	Value		*P* value	Value		*P* value	Value		*P* value	Value		*P* value

STT_Clinical Use_×STT _Supply Chain_, β (SE)	–.0547^b^ (0.015)	<.001	—^c^		—	—		—	.0715^d^ (0.033)		.03	—		—	—		—
STT_Clinical Use_×mobile IT, β (SE)	—	—	.0777^b^ (0.015)		<.001	—		—	—		—	.0012 (0.027)		.96	—		—
STT_Clinical Use_×HIE^e^, β (SE)	—	—	—		—	–.0051 (0.018)		.78	—		—	—		—	–.0411 (0.041)		.31
Hospital control	Yes	—	Yes		—	Yes		—	Yes		—	Yes		—	Yes		—
Patient control	Yes	—	Yes		—	Yes		—	Yes		—	Yes		—	Yes		—
Time and hospital fixed effects	Yes	—	Yes		—	Yes		—	Yes		—	Yes		—	Yes		—
Observations, n	662,874	—	662,874		—	662,874		—	186,496		—	186,496		—	186,496		—
Patients, n	262,562	—	262,562		—	262,562		—	80,130		—	80,130		—	80,130		—
Number of hospitals	40	—	40		—	40		—	19		—	19		—	19		—

^a^Main effects are included. Constant is included. Robust SEs were applied, and the errors were further clustered at the patient level to account for possible within-patient correlations. To ensure consistency in the analysis, 2 hospitals were excluded as their affiliation status varied across different years.

^b^*P*<.001.

^c^Not available.

^d^*P*<.05.

^e^HIE: health information exchange.

Table 3 presents the results regarding the influence of disease type on these joint effects. We identify that for patients whose primary diagnosis is a chronic disease, both the interaction effects between STT for clinical use and that for supply chain management and between STT for clinical use and mobile IT were associated with a lower 30-day readmission risk. For patients whose primary diagnosis is an acute disease, both the interaction effects between STT for clinical use and mobile IT and between STT and HIE were associated with a lower 30-day readmission risk. Consistent with previous literature, patients with chronic disease require long-term monitoring and ongoing care management. Such needs can be significantly aided by the combined use of STT for clinical use and supply chain management or mobile IT, which allows for continual updates, tracking, access, and analysis of medications and patients’ clinical information [6,25,34]. For patients with acute conditions that need immediate intervention, the benefit of HIT lies in its rapid and accurate information processing [25,34]. This advantage is particularly noticeable when STT for clinical use is combined with either mobile IT or HIE. This synergy enables instant access and capturing of a patient’s clinical information within and across health care facilities, thereby streamlining the decision-making processes and improving clinical outcomes.

**Table 3:** The role of disease type on the effects of smart track technology (STT) for clinical use^a^. Logistic regression was applied to assess the impact of health ITs (HITs) on 30-day readmission risk. The data sample in models 1 to 3 included 428,174 in-patient admissions with chronic conditions and models 4 to 6 included 450,948 in-patient admissions with acute conditions in the states of New York and Florida in the United States between 2014 and 2015.

Variable	Chronic conditions									Acute condition							
	1			2			3			4			5			6	
	Value	*P* value	Value		*P* value	Value		*P* value	Value		*P* value	Value		*P* value	Value		*P* value
STT_Clinical Use_×STT_Supply Chain_, β (SE)	–.0448^b^ (0.016)	.006	—^c^		—	—		—	–.0211 (0.017)		.21	—		—	—		—
STT_Clinical Use_×mobile IT, β (SE)	—	—	.0470^b^ (0.015)		.002	—		—	—		—	–.0577^d^ (0.015)		<.001	—		—
STT_Clinical Use_×HIE^e^, β (SE)	—	—	—		—	–0.0080 (0.015)		.59	—		—	—		—	–.0300^f^ *(0.014)*		*.04*
Hospital control	Yes	—	Yes		—	Yes		—	Yes		—	Yes		—	Yes		—
Patient control	Yes	—	Yes		—	Yes		—	Yes		—	Yes		—	Yes		—
Time and hospital fixed effects	Yes	—	Yes		—	Yes		—	Yes		—	Yes		—	Yes		—
Observations, n	428,174	—	428,174		—	428,174		—	450,948		—	450,948		—	450,948		—
Patients, n	233,608	—	233,608		—	233,608		—	245,567		—	245,567		—	245,567		—
Hospitals, n	61	—	61		—	61		—	61		—	61		—	61		—

^a^Main effects are included. Constant is included. Robust SEs were applied, and the errors were further clustered at the patient level to account for possible within-patient correlations.

^b^*P*<.01.

^c^Not available.

^d^*P*<.001.

^e^HIE: health information exchange.

^f^*P*<.05.

## Discussion

### Principal Findings

This study is the first of its kind to use complementarity theory to comprehensively examine the joint effects between STT for clinical use and that for supply chain management, between STT for clinical use and mobile IT, and between STT for clinical use and HIE on 30-day all-cause readmission risks using a large in-patient longitudinal dataset across 2 years from US-based hospitals. Our results revealed that while individual HIT implementations have varying impacts on the readmission risk, the combined use of HITs often yields a more notable effect on the reduction of readmission instances. This highlights the importance of an integrated approach to HIT deployment in health care for readmission reduction.

Furthermore, the empirical results of this study provide evidence for the *pooled complementarity* effect between STT for clinical use and STT for supply chain management. The results also provide evidence for *symbiotic complementarities* between STT for clinical use and mobile IT and between STT for clinical use and HIE. Specifically, this study has quantified the complementarity effects that arise from the combined use of HITs in US hospital settings and, thus, significantly contributes to the literature on the complementarity effects of HITs. Specifically, our findings enrich the current HIT complementarity literature by suggesting the potential benefits of HIT complementarity in both the IS and health informatics fields. Consistent with the previous studies that identified complementarity between different types of HITs [19,24,25], this study also identified a pooled complementarity effect between STT for clinical use and STT for supply chain management. The researchers also identified symbiotic complementarity effects between STT for clinical use and both mobile IT and HIE, respectively. As the initial study that quantifies the pooled complementarity effect and symbiotic complementarity effects of STT for clinical use, this study opens up future research toward the collective impacts of HITs on the strategic growth of hospitals, technological advancement and investments, deliverables, and other patient-centered care issues.

This study further illuminates the role of hospital affiliation and disease type on the efficacy of combining the use of STT for clinical purposes with STT for supply chain management, mobile IT, and HIE. The study findings highlight the differential impact of this integration, revealing that for hospitals within a health system and patients with chronic conditions, the joint use of STT for clinical applications with STT for supply chain management and mobile IT was associated with a decrease in the 30-day readmission risk. Meanwhile, hospitals outside the health system experienced an increase in 30-day readmissions when STT for clinical use was combined with STT for supply chain management. For patients with acute diseases, the bundling of STT for clinical use with mobile IT and HIE was beneficial in reducing 30-day readmissions. Thus, further research is needed to analyze the joint effects of HITs in various health care settings and patient populations. Further studies are required to explore these complementary HIT interactions and to develop robust guidelines for the effective use of the aforementioned HIT interventions in different health care contexts.

This research has several practical implications that could facilitate the joint adoption of state-of-the-art technologies such as STT for clinical use. First, before the introduction of any collective HIT systems, it is critical to assess diverse perspectives on hospital management, operations, clinical workflows, and patient care rather than assuming that the joint implementation of digital innovation in health care would always lead to optimal outcomes. Second, health care practitioners and hospitals should have a comprehensive view of the adoption of STT for clinical use and other HITs and their integration for critical outcome evaluations in US hospitals. This study provides unique empirical evidence on the complexity of various types of HIT integrations and the interoperability of various technologies. More specifically, this study found both pooled and symbolic complementarity effects in understanding the joint use of STT for clinical use, STT for supply chain management, mobile IT, and HIE in hospital settings. Third, this study has managerial implications on how hospitals could contextualize their HIT implementation and integration to maximize the benefits by implementing and using STT for clinical use in specific contexts and use cases. In other words, hospitals need to consider their unique use context, such as their hospital affiliation status and the types of diseases most commonly treated in their hospitals, while deciding how to implement HIT systems. For example, patients with chronic diseases, who demand continual monitoring and comprehensive care, can significantly benefit from the integration of STT for clinical use with that for supply chain management or mobile IT. These tools facilitate real-time monitoring, efficient medication tracking, and offer unhindered access to important patient information. In contrast, patients diagnosed with acute conditions requiring prompt medical interventions can derive benefits from the joint use of STT for clinical use and either mobile IT or HIE. Practitioners need to be mindful to assess the complementarity effects of multiple HITs for streamlining their specific clinical workflows and investing in emerging HITs with the potential to significantly enhance hospital operational efficiency and patient outcomes.

### Limitations

There are a few limitations in this study, creating new opportunities for future work. First, this study is limited to in-patient settings. Therefore, future studies should be conducted to explore whether the reported joint effects remain true in other health care contexts. Second, the retrospective data are extracted from a 2-year period and is based out of New York and Florida. Therefore, further research should be conducted with data collected from more US states that spans a longer period to further understand the long-term joint effects and change trajectories of STT and other relevant HITs on health outcomes [52]. Third, while pooled and symbiotic complementarities among the HITs were identified, the underlying mechanisms were not explored due to the data limitations of this study. Therefore, future research should further investigate the mechanisms of generating these complementarity effects. For instance, future studies can explore ways to integrate and process clinical data as it flows among different HIT systems. Fourth, this study is primarily centered on the complementarity effects of various HITs. However, previous studies also pointed out that using HITs can also lead to unintended consequences [53,54], which are rarely explored especially when dealing with multiple HITs. Therefore, we call for future studies to focus on the unintended outcomes stemming from interoperability issues among different technologies. Fifth, while this research primarily investigates the complementarity effects of HITs on the 30-day readmission risk, other patient-level and hospital-level health outcomes, such as health care costs, patient in-hospital length of stay, and mortality risk, should be examined in this context.

### Conclusions

This study aims to investigate the joint effects of STT for clinical use with STT for supply chain management, mobile IT, and HIE on the risk of 30-day all-cause readmission. By analyzing a large in-patient dataset from multiple sources across 61 hospitals in Florida and New York in the United States from 2014 to 2015, this study provided the first empirical examination of the complementarity effects of these HIT technologies in a longitudinal setting. The results showed a pooled complementarity effect between STT for clinical use and STT for supply chain management. This study also showed symbiotic complementarity effects between STT for clinical use and mobile IT and between STT for clinical use and HIE. Moreover, hospital affiliation and disease type significantly influenced the HIT complementarity effects. This study is the first evidence-based longitudinal study to quantify the complementarity effects of STT for clinical use with STT for supply chain management, mobile IT, and HIE. Therefore, it significantly contributes to an emerging stream of HITs through a complementarity theory lens in both the IS and health informatics fields. It also provides practical implications to be informative to managerial decisions by considering the complementarity effects of various HITs in a hospital setting.

## Appendix

Multimedia Appendix 1 Technology items in smart tracking technology for clinical use and electronic health record.

Multimedia Appendix 2 Summary statistics.

Multimedia Appendix 3 Model specifications.

## Abbreviations

AHA: American Hospital Association
EHR: electronic health record
HCUP: Healthcare Cost and Utilization Project
HIE: health information exchange
HIT: health IT
IRB: institutional review board
IS: information system
STT: smart tracking technology

## Data Availability

Access to the datasets used in this study is limited due to use restrictions imposed by the American Hospital Association (AHA) data use agreement and the Healthcare Cost and Utilization Project data use agreement. The datasets can be accessed with permission from the AHA [39] and Agency for Healthcare Research and Quality [38].
