# Supplementary material for: Harnessing the Power of Complementarity Between Smart Tracking Technology and Associated Health Information Technologies: Longitudinal Study
Source: JMIR Form Res. 2024 Oct 1;8:e51198. doi: 10.2196/51198 (PMC11480677; doi:10.2196/51198)
Supplement: Multimedia Appendix 1 [file formative_v8i1e51198_app1.docx]

# **Appendix 1** **Technology Items in STT for Clinical Use and EHR**

STT for clinical use and EHR implementation level for each itemized technology is measured on a six-point scale, where a rating of 1 to signify complete implementation across all units, ratings of 2 to 5 to denote partial implementation at varying degrees, and a rating of 6 corresponds to no implementation and no intentions to adopt the technology. To calculate the HIT implementation level for each clinical function, we applied a coding scheme to differentiate complete implementation from partial or no implementation. Specifically, responses categorized as 1 remain unchanged, while those between 2 and 6 are recoded as 0.

Part I: Technology Items in STT for Clinical Use (4 technology items in total)

Bar coding or RFID for closed‐loop medication tracking:

a. Medication administration

b. Patient verification

c. Caregiver verification

d. Pharmacy verification

Part II: Technology Items in EHR (24 technology items in total)

EHR contains four main functions electronic clinical documentation, results viewing, computerized provider order entry, and clinical decision support systems. The detailed items for each function are listed below:

1. Electronic clinical documentation

a. Patient demographics

b. Physician notes

c. Nursing notes

d. Problem lists

e. Medication lists

f. Discharge summaries

g. Advanced directives (e.g., DNR)

2. Results viewing

a. Laboratory reports

b. Radiology reports

c. Radiology images

d. Diagnostic test results (e.g., EKG report, Echo report)

e. Diagnostic test images (e.g., EKG tracing)

f. Consultant reports

3. Computerized provider order entry

a. Laboratory tests

b. Radiology tests

c. Medications

d. Consultation requests

e. Nursing orders

4. Clinical decision support systems

a. Clinical guidelines

b. Clinical reminders

c. Drug allergy alerts

d. Drug-drug interaction alerts

e. Drug-lab interaction alerts

f. Drug dosing support
