# Supplementary material for: Harnessing the Power of Complementarity Between Smart Tracking Technology and Associated Health Information Technologies: Longitudinal Study
Source: JMIR Form Res. 2024 Oct 1;8:e51198. doi: 10.2196/51198 (PMC11480677; doi:10.2196/51198)
Supplement: Multimedia Appendix 2 [file formative_v8i1e51198_app2.docx]

# **Appendix 2** **Summary Statistics**

| **Variable name** | **Mean** | **std.** | **Min** | **Max** |
| --- | --- | --- | --- | --- |
| HIT Variables |  |  |  |  |
| STT_Clinical_ | 2.7636 | 1.4228 | 0 | 4 |
| STT_Supply Chain_^†^ | 0.5100 | 0.4999 | 0 | 1 |
| Mobile IT^†^ | 0.7065 | 0.4554 | 0 | 1 |
| HIE^†^ | 0.6836 | 0.4651 | 0 | 1 |
| EHR | 21.3569 | 3.5383 | 0 | 24 |
| Hospital Variables |  |  |  |  |
| No. of beds | 847 | 752 | 40 | 2654 |
| Teaching^†^ | 0.4762 | 0.4994 | 0 | 1 |
| Not for profit^†^ | 0.7947 | 0.4039 | 0 | 1 |
| Health System^†^ | 0.7702 | 0.4207 | 0 | 1 |
| Metro Hospital^†^ | 0.9761 | 0.1527 | 0 | 1 |
| FL indicator^†^ | 0.4858 | 0.4998 | 0 | 1 |
| Patient-Admission Variables |  |  |  |  |
| Discharge age | 65.3065 | 17.5524 | 18 | 109 |
| Female^†^ | 0.5184 | 0.4997 | 0 | 1 |
| White^†^ | 0.6031 | 0.4892 | 0.6031 | 0.4892 |
| Black^†^ | 0.1704 | 0.3760 | 0.1704 | 0.3760 |
| Hispanic^†^ | 0.1378 | 0.3447 | 0.1378 | 0.3447 |
| Asian/Pacific Islander^†^ | 0.0166 | 0.1279 | 0.0166 | 0.1279 |
| Native American^†^ | 0.0021 | 0.0462 | 0.0021 | 0.0462 |
| Race: Other^†^ | 0.0699 | 0.2550 | 0.0699 | 0.2550 |
| Medicare^†^ | 0.6300 | 0.4828 | 0 | 1 |
| Medicaid^†^ | 0.1598 | 0.3664 | 0 | 1 |
| Private insurance^†^ | 0.1512 | 0.3583 | 0 | 1 |
| Chronic primary diagnosis^†^ | 0.4870 | 0.4998 | 0 | 1 |
| No. of comorbidity | 3.3680 | 1.9607 | 0 | 15 |
| No. of chronic diagnoses | 6.8076 | 3.3197 | 0 | 25 |
| No. of diagnoses | 13.1759 | 6.3149 | 3 | 31 |
| No. of procedures | 1.9253 | 2.5717 | 0 | 31 |
| Emergency or urgent^†^ | 0.8791 | 0.3260 | 0 | 1 |
| Transfer Body System^†^ | 0.5577 | 0.4967 | 0 | 1 |
| No. of Visits | 2.5470 | 2.4815 | 1 | 68 |
| Days in Between Visits | 180 | 233 | 1 | 1456 |
| Disease types |  |  |  |  |
| Infectious and parasitic disease^†^ | 0.0795 | 0.2705 | 0 | 1 |
| Neoplasms^†^ | 0.0433 | 0.2036 | 0 | 1 |
| Endocrine, nutritional, and metabolic diseases and immunity disorders^†^ | 0.0495 | 0.2169 | 0 | 1 |
| Diseases of blood and blood-forming organs^†^ | 0.0233 | 0.1510 | 0 | 1 |
| Mental disorders^†^ | 0.0520 | 0.2221 | 0 | 1 |
| Diseases of the nervous system and sense organs^†^ | 0.0287 | 0.1671 | 0 | 1 |
| Diseases of the circulatory system^†^ | 0.1993 | 0.3995 | 0 | 1 |
| Diseases of the respiratory system^†^ | 0.1056 | 0.3073 | 0 | 1 |
| Diseases of the digestive system^†^ | 0.1118 | 0.3151 | 0 | 1 |
| Diseases of the genitourinary System^†^ | 0.0579 | 0.2335 | 0 | 1 |
| Diseases of the skin and subcutaneous tissue^†^ | 0.0237 | 0.1521 | 0 | 1 |
| Diseases of the musculoskeletal system^†^ | 0.0502 | 0.2184 | 0 | 1 |
| Congenital anomalies^†^ | 0.0011 | 0.0328 | 0 | 1 |
| Symptoms, signs, and ill-defined conditions^†^ | 0.0573 | 0.2325 | 0 | 1 |
| Injury and poisoning^†^ | 0.0925 | 0.2898 | 0 | 1 |
| Factors influencing health status and contact with health services^†^ | 0.0242 | 0.1537 | 0 | 1 |

†: Dummy variables with two values: 0 and 1
