# Supplementary material for: Harnessing the Power of Complementarity Between Smart Tracking Technology and Associated Health Information Technologies: Longitudinal Study
Source: JMIR Form Res. 2024 Oct 1;8:e51198. doi: 10.2196/51198 (PMC11480677; doi:10.2196/51198)
Supplement: Multimedia Appendix 3 [file formative_v8i1e51198_app3.docx]

# **Appendix 3** **Model Specifications**

${Readmission}_{ijt}=\beta_{0}+ \beta_{1}{STT\_ClinicalUse}_{j,t-1}+ \beta_{2}{STT\_SupplyChain}_{j,t-1}+ \beta_{3}{Mobile IT}_{j,t-1} + \beta_{4}{HIE}_{j,t-1}+ \beta_{5}{Hospital\_Control}_{jt}+ \beta_{6}{Patient\_Control}_{ijt}+Time\_Dummy+Hospital\_Dummies+ \varepsilon_{ijt}$ (1)

In Equation (1), *i* indexes a patient’s hospital visit, *j* indexes the hospital, and *t* indexes the time in year. The focal parameters of interest in this model are $\beta_{1}$, $\beta_{2},$ $\beta_{3}$, and$\beta_{4}$, which capture the main effects of STT for clinical use, STT for supply chain management, mobile IT, and HIE. ${Hospital\_Control}_{jt}$ represents hospital-level control variables. ${Patient\_Control}_{ijt}$ represents patient-admission level control variables. We also include $Hospital\_Dummies$in the model to account for the hospital fixed effects. We also include $Time\_Dummy$ to account for the time effect. $\varepsilon_{ijt}$ denotes the error term.

${Readmission}_{ijt}=\beta_{0}+ \beta_{1}{STT\_ClinicalUse}_{j,t-1}+ \beta_{2}{STT\_SupplyChain}_{j,t-1}+ \beta_{3}{Mobile IT}_{j,t-1} + \beta_{4}{HIE}_{j,t-1}+\beta_{5}{(STT\_ClinicalUse}_{j,t-1}*{STT\_SupplyChain}_{j,t-1}) + \beta_{6}{Hospital\_Control}_{jt}+ \beta_{7}{Patient\_Control}_{ijt}+Time\_Dummies+Hospital\_Dummies+ \varepsilon_{ijt}$ (2)

In Equation (2), the focal parameter is $\beta_{5}$, which captures the interaction effects of STT for clinical use and for supply chain management.

${Readmission}_{ijt}=\beta_{0}+ \beta_{1}{STT\_ClinicalUse}_{j,t-1}+ \beta_{2}{STT\_SupplyChain}_{j,t-1}+ \beta_{3}{Mobile IT}_{j,t-1} + \beta_{4}{HIE}_{jt}+\beta_{5}{(STT\_ClinicalUse}_{j,t-1}*{Mobile IT}_{j,t-1}) + \beta_{6}{Hospital\_Control}_{jt}+ \beta_{7}{Patient\_Control}_{ijt}+Time\_Dummies+Hospital\_Dummies+ \varepsilon_{ijt}$ (3)

In Equation (3), the focal parameter is $\beta_{5}$, which captures the interaction effects of STT for clinical use and mobile IT.

${Readmission}_{ijt}=\beta_{0}+ \beta_{1}{STT\_ClinicalUse}_{j,t-1}+ \beta_{2}{STT\_SupplyChain}_{j,t-1}+ \beta_{3}{Mobile IT}_{j,t-1} + \beta_{4}{HIE}_{j,t-1}+\beta_{5}{(STT\_ClinicalUse}_{j,t-1}*{HIE}_{j,t-1}) + \beta_{6}{Hospital\_Control}_{jt}+ \beta_{7}{Patient\_Control}_{ijt}+Time\_Dummies+Hospital\_Dummies+ \varepsilon_{ijt}$ (4)

In Equation (4), the focal parameter is $\beta_{5}$, which captures the interaction effects of STT for clinical use and HIE.
